# Sustainable Smart Industry: A Secure and Energy Efficient Consensus Mechanism for Artificial Intelligence Enabled Industrial Internet of Things

**DOI:** 10.1155/2022/1419360

**Published:** 2022-06-20

**Authors:** A. Sasikumar, Logesh Ravi, Ketan Kotecha, Jatinderkumar R. Saini, Vijayakumar Varadarajan, V. Subramaniyaswamy

**Affiliations:** ^1^Department of Electronics and Communication Engineering, Vel Tech Rangarajan Dr. Sagunthala R&D Institute of Science and Technology, Avadi, Chennai, India; ^2^SENSE, Vellore Institute of Technology, Chennai, Tamilnadu, India; ^3^Symbiosis Centre for Applied Artificial Intelligence, Symbiosis International (Deemed University), Pune, India; ^4^Symbiosis Institute of Computer Studies and Research, Symbiosis International (Deemed University), Pune, India; ^5^Ajeenkya DY Patil University, Pune, India; ^6^School of Computer Science and Engineering, University of New South Wales Sydney, Kensington, NSW, Australia; ^7^School of Computing, SASTRA Deemed University, Thanjavur, India

## Abstract

In recent years, the Internet of Things (IoT) has been industrializing in various real-world applications, including smart industry and smart grids, to make human existence more reliable. An overwhelming volume of sensing data is produced from numerous sensor devices as the Industrial IoT (IIoT) becomes more industrialized. Artificial Intelligence (AI) plays a vital part in big data analyses as a powerful analytic tool that provides flexible and reliable information insights in real-time. However, there are some difficulties in designing and developing a useful big data analysis tool using machine learning, such as a centralized approach, security, privacy, resource limitations, and a lack of sufficient training data. On the other hand, Blockchain promotes a decentralized architecture for IIoT applications. It encourages the secure data exchange and resources among the various nodes of the IoT network, removing centralized control and overcoming the industry's current challenges. Our proposed approach goal is to design and implement a consensus mechanism that incorporates Blockchain and AI to allow successful big data analysis. This work presents an improved Delegated Proof of Stake (DPoS) algorithm-based IIoT network that combines Blockchain and AI for real-time data transmission. To accelerate IIoT block generation, nodes use an improved DPoS to reach a consensus for selecting delegates and store block information in the trading node. The proposed approach is evaluated regarding energy consumption and transaction efficiency compared with the exciting consensus mechanism. The evaluation results reveal that the proposed consensus algorithm reduces energy consumption and addresses current security issues.

## 1. Introduction

The digitization transition gives expressive possibilities for the industry to grow creative and changing economic models and complex circular distribution networks. However, the information technology and transmission sector have a small impact on the environment; such a conversion has significant consequences for sustainability. It is vital to supply solutions in a resilient and comprehensive manner throughout their entire life cycle to meet the milestones set forth by the industrial revolution for sustainable development [1] and achieve the circular economy's goals. Three fundamental innovative models enable the long-term digitization of a smart circular economy: industrial IoT, edge-based computing, and artificial intelligence (AI).

The introduction of the context of big data and two dominant digital innovations, such as machine learning and the Internet of Things, has recently been experienced around the world. Whereas the Internet of Things establishes a network of interlinked systems, machine learning (AI) allows machines to mimic cognitive abilities. AI and the IoT can work together to allow a new potential technology called Artificial Intelligence of Things (AIoT). In general, AIoT intends to make IoT production effective, increase human-machine interactions, and improve data gathering and analysis capabilities. These innovative techniques are developed through intelligent system advances in hardware (e.g. edge devices and accelerators) and software (e.g. RTOS, digital twin, deep learning architecture); AIoT is becoming the real-time application. In recent times, AIoT has been adopted in many application areas such as smart home automation [2], industry automation, and smart cities [3].

**Figure 1:**
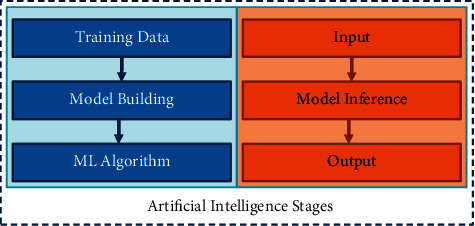
Artificial Intelligence two-stage process.

AIoT, as an AI-enabled framework, follows the standard training and inference approach [4] depicted in Figure 1. In the first stage, AI models utilise various machine learning techniques for training the data set. In the deployment of AIoT, training data have frequently gathered various data from IoT devices. Models are developed in the second step to arrive at conclusions from specific information. The two processes are commonly referred to as model development and Inferencing. A fundamental difficulty in AIoT is that the development of the model algorithm stage necessitates a large quantity of data and processing capabilities to produce the best AI models [5]. Yet, most IoT devices lack the necessary storage resources due to different constraints.

With the development of smart sensor technologies to integrate AI-based systems deployed in real-time applications, all data start from the era of the Industry 4.0 revolution [6]. Smart sensors are a topic that contributes to the enhancement of production and increased turnover in a variety of industries [7]. These advantages have been proven, especially when the technology available on the market is used effectively. However, AI applications can be harmful in some situations, causing major problems for the company in question. Furthermore, sensors can respond differently in different environments. They may give data of varying quality, which might misidentify the model decision and result in categorization failures if the model is not sufficiently stable. A significant effort and high costs are associated with an AI-based system developed to solve a single classification challenge, and a single misclassification scenario is costly.

The disadvantages of misclassification differ from one area to the next, based on a specific domain. On the one side, in the medical field, when a computerized diagnosis suggests that a person is sick, but in reality his health is not that poor [8]. Later, a doctor can verify and discover that the patient is, in fact, healthy. In the opposite situation, failing to recognize a sick patient and allowing him or her to continue without treatment is extremely harmful. Human safety is taken into account here; hence, high classification accuracy of greater than 99.99 per cent is essential. On the other hand, we permit marginally greater categorization errors for most industrial settings that do not endanger people's safety.

Based on various research studies in the agricultural field, the work of Xiong et al. [9], as well as Wossen et al. [10], validated that the cost of a misclassification error varies based on whether it is a false-negative or false-positive error in terms of financial and material loss. Various fault prediction approaches have been developed in the literature [11] to achieve low classification error rates. Other Support Vector Machine (SVM)-based approaches for minimizing misclassification situations have been proposed [12]. These approaches require a significant amount of training data, that is, data that contain classified information. This step is still challenging and time-consuming, particularly when working on new software programs that lack previous defect data.

Furthermore, time is a critical issue because these received much attention in the post-evaluation of the classification stage. Achieving a low classification error rate with the lowest possible risks is essential. Even more model re-training must be conducted after the prediction of the misclassification impact based on reference data, as demonstrated by Xiong, Y. and Zuo and Xiong in their paper [13], where they investigate the effect of misclassification errors to train a cost-effective neural network with different expense proportions. This method is time-consuming and requires an online training variable change despite the positive findings. There are also additional types of studies interested in evaluating sensor data online. In their study [14], Song and Deng use proof theory and intuitionistic fuzzy to continuously assess the trustworthiness of sensor data. As a result, the system can assign a fair dependability factor to sensors that give contradictory data. On the other hand, the proposed system design is extremely sophisticated and necessitates a good mathematics background.

Combining data from several sources yields significant benefits for decision-making and framework management, primarily in terms of increased trust and better-resolved system information. However, deciding on appropriate sensors to integrate for a specific task is difficult. Time and money are generally spent on respectful considerations. Instead, we present a clever AI-based solution to advanced sensor fusion that determines multiple sensor data streams based on the individual requirements, situations, and tasks. We recommend using AI automation as a pre-evaluation method in particular. This AI-based sensor assessment and clever fusion using interpretable frameworks technique can be easily applied to a wide range of sensor fusion systems.

As a result, the model's interoperability allows the candidate to follow the decision-making activity. In addition, the suggested method can deny a request if the model is unsure about a decision. We demonstrate how to create a bi-functional system that incorporates both aspects. We concentrate on both the dynamic and static pre-evaluation of the system. A dynamic pre-evaluation evaluates sensor inputs during the classifier training phase. In contrast, a static pre-evaluation is done offline after the classifiers have been taught but before they are used in manufacturing lines. The developed system enables the evaluation of each sensor in terms of its data contribution to a predetermined categorization assignment and the hardiness of this information based on various external conditions.

Artificial intelligence (AI) has been hailed as a cure for a slew of problems in various industries [15]. It can upend old business models by opening new ones [16–18]. AI applications in the industrial business promise unique services in addition to efficiency improvements [19]. Enhancing goods with data-driven solutions is a crucial revenue generator in marketplaces with shrinking margins. It allows companies to stand out from competitors, especially in environments with ubiquitous nondata-driven services [20]. As discussed in this research [21], AI technologies are particularly valuable for commercial comprehensive supplier marketing strategies. Full-service providers (FSPs) retain ownership of intellectual products (e.g., industrial systems) and offer their use as a service in the manufacturing sector. FSP customers gain from converting procurement expenses into usage- or time-based costs, as well as the elimination of operational costs and the transfer of property control risks to the FSP. The FSP, on the other hand, gains from improved client loyalty [22] and additional revenue streams by embracing innovative payment formats. AI applications have the potential to boost the profitability of FSP marketing strategies by lowering maintenance costs and improving the availability of products or durability [23].

On the other hand, most AI applications use statistical approaches to training as part based on information [24]. These predictions allow for categorization that helps with various industrial applications and services. Cost-effective predictive maintenance (PM) and computerized predictive quality assurance (PQ) are two examples of such applications. As a result, the categorization algorithms that FSPs use must add value to consumers–i.e., service recipients (SRs)–while still being profitable.

Although most machine learning techniques are designed to turn even complicated cognitive issues into a binary classification [25], we look into classification techniques. Furthermore, the statistical structure of today's AI applications renders classifications inaccurate; nonetheless, studies show that up to 30% of decision-makers lack a basic knowledge of AI. Firms wanting to reinvent data-driven solutions based on categorization techniques must account for this imperfection in the construction of service-level agreements (SLAs) to obtain the promised benefits. As a result of the poor predictive power (PP), misclassifications resulted in breakdowns and decreased service levels. The FSP, for example, may be required to pay the SR for a lesser service level, resulting in additional costs.

Similarly, fluctuating service standards could impact an FSP's revenue. The revenue fluctuates regarding the payment system according to the PP-dependent quality of service. Low PP has little effect on a subscriber payment schedule, which provides FSPs with consistent revenue. On the other hand, a high PP allows FSPs to grow income by increasing service levels in utilization payment models. On the other hand, Low PP reduces the level of services provided and, consequently, income. The interaction of PP and different payment arrangements might positively or negatively impact the FSP's estimated net present value (NPV) [26]. As a result, FSPs must use an economic calculus to weigh the risks and benefits of using classification algorithms to pick payment arrangements.

### 1.1. Contribution

In Industrial automation applications, integrating blockchain, artificial intelligence, and big data constitutes the core technologies that allow dynamic data transmission. Moreover, integrating these technologies provides many features in addressing the challenges related to security, such as transparency, privacy, ensuring ownership rights, decentralization, and so on [27]. The integration of blockchain and artificial intelligence, on the other side, is still being investigated. More research studies have recommended artificial intelligence adoption using a simplified distributed system, with a focus on decentralized authentication. These research studies have failed to develop an artificial intelligence-based big data security model. Furthermore, the blockchain is not employed for big data analytics to overcome the risk of handing dynamic data into the system [28]. Instead, researchers implemented a blockchain model incorporated with distributed ledger for secure transaction processes in the industries.

In this manuscript, we introduce an improved DPoS-based consensus algorithm to increase the data transaction speed, decentralized control, and data security for IIoT networks. The novelty of developed consensus mechanism for industrial applications is as follows:

- To resolve the centralized security problems of IIoT, we proposed an improved DPoS consensus algorithm based on honor delegates for real-time applications.
- To accelerate IoT block creation, nodes use an improved DPoS to reach a consensus for selecting delegates and store block information in the trading node.
- Due to the demanding needs of enabling technologies in industrial applications, the data transmission and energy consumption are challenging tasks. To overcome these issues, we introduce delegates and honor delegate nodes–based consensus algorithm for AI-enabled IioT.

## 2. Related Work

**Figure 2:**
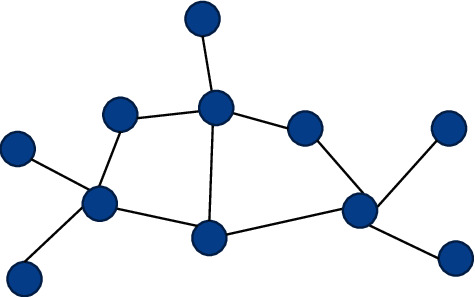
Graph-based IoT node generation for industrial applications.

In this section, we describe the basic mechanism of artificial intelligence, big data, and blockchain for industrial application and how consensus mechanism-based AI change industrial IoT. Blockchain, data science, and AI are the enabling technologies for industrial applications. Blockchain is mainly focused on the distributed ledge and decentralized framework for real-time applications. At the same time, data science is used for providing dynamic information and AI is employed for analyzing and classifying the sensor data in IoT applications. These innovative techniques are allowing machines to make decision and provide intelligent services.  Figure 2 shows the IoT node interconnection for industrial applications.

### 2.1. Artificial Intelligence and Big Data Overview

Big data has been one of the most prominent research topics in recent years. Because of its huge volume, rapid velocity, and heterogeneous diversity, it differs from regular data. These traits of volume, velocity, and variety are known as the 3 Vs of big data. Later, the list was expanded to include two more Vs: value and veracity. As a result, all data that are of substantial quantity (volume), generated at a high rate (velocity), and diverse in nature (organized, semi-organized, or unorganized) are referred to as big data (variety). The value of big data analytics is that it incorporates the fourth V (value) into its qualities, making it a valuable asset to the company.

Big data analytics is a technique for analyzing large amounts of data and turning it into useful information by employing cutting-edge statistical, analytical, logistic, or artificial intelligence methods. The 3 Vs of big data, on the other hand, introduce a new set of obstacles, such as collecting, storing, exchanging, organizing, processing, analyzing, and visualizing such large amounts of data at rapid speeds [29]. Various frameworks have been built to manage large data for successful analytics in various applications for this purpose.

The digital reproduction of three primary cognitive abilities: training, thinking, and self-correction, is known as artificial intelligence (AI). Digital learning is a set of principles applied as a predictive algorithm that transforms real-world historical data into useful information [30]. The purpose of digital reasoning is to select the best rules for achieving a specific goal. Digital self-correction, on the other hand, is the continuous process of accepting the results of reinforcement learning. This approach is followed by every AI model in order to create a smart technology that can accomplish a task that would ordinarily consume a lot of time.

Machine learning, deep learning, data analysis, and principle techniques are used in the majority of intelligent systems, while reasoning and experience and understanding methods are used in others [31]. Machine learning and deep learning are two AI methods that are commonly employed nowadays. The distinctions among artificial intelligence, machine learning, and deep learning methods are frequently misunderstood.

Machine learning is a type of artificial intelligence (AI) that looks for certain trends in past data to help with decision-making. The more data we gather, the more precise is the learning process (eliminate the term big data). Machine learning can be classified into three types based on the decision process. First, supervised learning, wherein sets of data containing labelled outputs are accepted in required amounts to practice a model for categorization or future projections. Second, unsupervised learning is a type of machine learning that works with unstructured sets of data which are used for clustering and sorting. Finally, reinforcement learning collects data recordings with no labels but delivers response to the intelligent agent once specified actions take place. Linear regression, decision tree, and SVMs are the examples of supervised machine learning algorithms [32]. *K*-means and hierarchical cluster analysis fall under the unsupervised learning [33]. Lastly, Monte Carlo learning and Q-learning comes in the categories of reinforcement learning techniques [34]. Deep learning is a data mining technique inspired by the biological neural network and utilising one or more hidden units of artificial neurons. The historical data are handled repeatedly by several layers during the learning process, creating links and continuously weighting the neuron inputs for best results.

### 2.2. Relationship between Artificial Intelligence, Big Data, and IIoT

Real-time surveillance of physical equipment, indoor asset management, and outdoor asset management are just a few of the novel opportunities enabled by new smart sensors and IoT deployments in industrial ecosystems [35]. By integrating the physical environment to its virtual picture, IoT devices promote the real-time data gathering required for the production of a digital model of the physical component and permit the enhancement and servicing of the physical component (using smart devices). Because the IoT data indicated above is large in size, big data analytics can be useful in the building of an effective technology.

**Figure 3:**
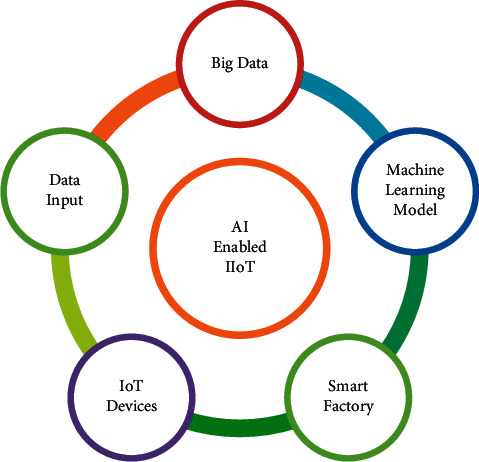
AI enabled IIoT network integration.

The reason for this is that industrial activities are extremely complicated, making early detection of possible issues difficult using conventional methods. Such issues, on the other side, may be easily retrieved from collected data, bringing productivity and expertise to industrial applications. However, in the industrial and technological realms, handling this massive volume of data necessitates complex approaches, structures, platforms, technologies, and algorithms. In a digital twin setting, for example, Zhang et al. [36] suggested a big data analytic system for smart account auditing and maintenance. The relationship between AI, big data, and IIoT is depicted in Figure 3.

Cloud technology is frequently the perfect platform for processing and analyzing large amounts of data [37]. Furthermore, only by using AI technologies on the obtained data how would an intelligent digital system be created. In a nutshell, the IoT is used to collect large amounts of data from the physical world. The data are then placed into an AI model to create a digital twin. The developed digital system can then be used to improve other industrial processes.

### 2.3. Research Challenges and Security Issues

The growing acceptance and accessibility of blockchain, as well as the use of IoT, data science, and AI innovations, has broadened the research problems of blockchain. These difficulties are divided into four categories.

#### 2.3.1. Data Collection

Data collecting from a physical device, data combining, and data exchanging with the associated blockchain are all made easier by the IoT. This procedure has the potential to be rather costly. It is possible that the digital ledger will be more expensive than the asset itself, in which case it will not be worthwhile to build the digital system. The acquired data, on the other hand, is huge, fragmented, unorganized, and noisy. As a result, more data processing is necessary to guarantee its optimal usage.

We need to use data cleaning procedures, as well as organize, rearrange, and homogenize the data. Furthermore, keeping such a massive volume of data under control is a huge task. Furthermore, the fundamental machine learning techniques require a specific quantity of data for training reasons in order to enhance the reliability of the blockchain model.

#### 2.3.2. Challenges in Big Data

The rapid use of IoT technology in the industries has resulted in massive volumes of monitoring (sensor) data being generated. To this goal, improved infrastructures, foundations, platforms, techniques, and strategies are needed to represent, preserve, share, analyze, and evaluate the raw data in big data and analytics. Edge and cloud services platforms could also be used to handle digital twin related data. Edge computing, in particular, allows for dispersed computation at the network's edge, with collective analysis taking place in the cloud. However, data processing on the cloud may result in a longer response time.

#### 2.3.3. Analysis of Raw Data

As described in the literature, artificial intelligence–based techniques for big data played an important role in industry for decision-making. However, choosing a certain model from hundreds of machine learning with unique settings is difficult. To various applications and data sources, each intelligence has various levels of accuracy and efficiency. On the other hand, accuracy might have a negative impact on efficiency. As a result, choosing the proper optimization algorithm and functionalities is difficult depending on the motivation and implementation of industry automation. Furthermore, there are less realistic deployments of intelligence for industry 4.0 revolution in the literature, which adds to the difficulties.

#### 2.3.4. Challenges in Privacy and Security

Some manufacturing sectors, such as sensor data, product-related information, and human management ledger are deemed sensitive and may demand strict security and privacy guarantees. First, because IoT devices are involved in digital twinning, the privacy of the fundamental communication systems must be prioritized. Furthermore, the enormous amount of asset-related data must be securely held to avoid data theft from both inside and outside attacks.

### 2.4. Need of Blockchain Technology in IIoT

In recent times, there has been a lot of studies into the privacy and security of interaction among IoT devices. Blockchain technology is a new use in IIoT networks, and its effective deployment has been the focus of much research. The IIoT benefits from the blockchain's decentralization, data integrity, cryptography privacy, fault tolerance, data security and identification, and consensus mechanism [38]. Several research studies compared popular blockchain platforms, including cryptocurrency, Ethereum [39], Hyperledger-Fabric [40], and IOTA [41], and discussed the advancement of smart contracts and its practicability in the industry, IOTA offers free transactions designed specifically for device to device communication, but it lacks the maturity of Ethereum and Public blockchain.

Blockchain technology has clearly evolved in people's perception as scientific research and innovations have progressed, and it has become a topic of studies by scholars and researchers. Industry and academics are paying growing attention to it. People have recognised the one-of-a-kind extraordinary development that distributed ledger may bring about and have committed in the development on business elements such as banking, healthcare, and traceability. The distributed system is speeding up the maturity and industrial adoption of blockchain technology. Presently, China is creating its own blockchain technology; the competitive market structure and the separate copyright system are being developed [42].

At the same time, relevant techniques and developed ecology are integrating sectors, such as energy, healthcare, and agriculture. The decentralized security is a new kind of innovative platform in that digital information such as random data blocks are used to authenticate user and provide the data privacy through consensus algorithm. The blockchain technology is implemented to ensure the security and privacy of data transmission between nodes.

### 2.5. Consensus Algorithm for Industrial Applications

A consensus algorithm is a collection of rules governing how a decentralized network is supposed to work. These principles outline the basic roles of various parts, how they interact, and the criteria that must be met in order for them to function correctly. A consensus algorithm specifies the rules that must be followed in order to establish an agreement, as well as the procedures that should be done under what situations. The proof of work (POW) technique states that as long as such a node could generate a block which adheres to the desired value, the entire network can verify it [43]. In a distributed system, a consensus algorithm is a technique for resolving data synchronisation between nodes that do not trust each other.

PoX (proof-of-X) decision techniques for blockchain systems without authorities have recently emerged and developed, with all techniques focusing on network transactions [43]. However, because there is no agreement, transaction verification is delayed, which is incompatible with most dynamic IIoT devices that demand real-time validation. The Equihash method [45] is a proof-of-work (PoW) agreement technique based on the generalised birthday dilemma in which a fundamental cryptography implementation is difficult. This is a memory-dependent consensus technique that sets the burden based on the nodes' storage sizes. It requires a lot of storage to provide evidence, but it can achieve quick confirmation. Although this design enhances the cost-effectiveness of ASIC devices, the application's security has yet to be validated.

The Ouroboros techniques deployed in Ref. [46] is a distributed system-based proof of stake (PoS) consensus model. The techniques develop a tight security guarantee consensus procedure and push the PoS consensus process via a reward system. This reward is to confirm that nonmalicious devices maintain a nash equilibrium and also prevent security breaches affected by selfish block creation. Da Xu and Viriyasitavat [47] combined the PoW and PoS consensus concepts for security transactions. The PoW method is utilised for the acquisition of tickets in the early stages of the process. When the blockchain system has acquired sufficient assets, the PoS algorithm is utilised to ensure the network's long-term safety. The PoS algorithm provides direct correlation between coin age and time that is converted to an exponentially decaying rate. This approach helps the rate of growth of coin age to reach zero over the period of time and prohibits the accumulation of money. However, the approach increases computation time and necessitates a large amount of network memory size.

Based on the original Paxos algorithmic concept, Moraru et al. [48] created EPaxos consensus method. Creating the dependency, accepting the request, and completing the phase are the three stages of the method. Each proposition has characteristics, such as gathering and pattern numbering, in addition to the intrinsic data. To establish the execution order of competing proposals, the ideas of a quick stream, slow stream, and dependency graph are presented. Only implementation situations with few or no conflicts are acceptable for the technique.

For the distributed ledgers, Sousa et al. [49] introduced the Byzantine fault-tolerant consensus protocol. To model the continuity of Byzantium fault-tolerance, this protocol employs a probability remuneration network. Although the protocol offers some benefits in terms of transmission capacity and faster transaction time, the execution flow could be improved. On the basis of credit, Yeow et al. [50] suggested an enhanced practical Byzantine fault-tolerant (PBFT) consensus protocol. The consensus protocol was enhanced, credit assessment predicated on a coalition chain was created, and the system was brought into a feedback loop by including a lightweight integrity method. The checkpoints protocol was changed to allow devices to enter and depart the network on demand, increasing the platform's adaptability.

Lin et al. [51] developed a ring signature-based modified PBFT consensus technique. The PBFT technique, the ElGamal cryptographic signature encryption method, and the ring signature concept were all presented. The efficiency and secrecy of a ring signature technique based on the ElGamal technique were then investigated. The ring signature strategy was optimized to enhance the PBFT computation signature and validation procedure, allowing nodes to enter and depart the network continuously. The suggested solution surpassed the original PBFT computation fault-tolerant percentage.

Delegated Proof of Stake (DPoS) was created by Larimer and deployed initially for the BitShares project [52]. The DPoS consensus process is separated into two parts: the first is the election of witnesses (block creators), and the second is the generation of blocks. Witnesses are simply authorized for confirming the transactions, validating the signature, and timestamping it; they are not allowed to trade. They each produce one block every three seconds, and if a witness fails to perform the task within the time limit, it is ignored and substituted by the next one. Each network node has the ability to vote for its own dedicated witness, and the more smart contract stakes he or she has, the more likely he or she is to be a witness. However, because of the core method whereby each witness node generates blocks in succession, the identification of the witness is already established and stable, making the distributed ledger network more open to fraud assaults.

Furthermore, achieving fairness by solely employing DPoS is challenging, as it will only allow those with more resources to become voters. In addition, while employing the PoW alone, the block period is around 10 minutes, wasting a significant amount of computer and energy resources. For this third issue, this paper proposes an honor mechanism akin to that used in reputation to select the delegate node that causes the consensus to be harmed and replace them.

There are two types of nodes in the consensus algorithm, according to our findings. The delegate node, for example, is a node that creates or validates transactions and contributes in the consensus mechanism. The honor delegate node, on the other hand, serves as a contender for the delegate node and is used to replace it if it fails. These two types of nodes are termed as delegate nodes for conforming consensus mechanism.

### 2.6. Proposed Blockchain-Based Consensus Mechanism for AI-Enabled IIoT

To resolve the security and privacy issues of AI and enable IIoT, considering the distributed ledge of sensor information, we proposed an improved DPoS consensus mechanism based on honor voting system for industrial application. We construct a consensus mechanism–based ledger for sensor data storage in IoT device system solutions for smart industrial automation because of the transparency and data integrity of blockchain. Data cannot be changed by distant attackers wanting to quickly get into the device for harmful modification. Since IoT sensor data, such as identification, password protection, application settings, and behavioral records, may be safely kept in distributed ledger, in this paper, we employed an improved DPoS algorithm to create a consensus for producing blocks including sensor information, which speeds up block generation.

### 2.7. Improved DPoS Consensus Algorithm

The most appropriate delegates cannot be picked for block formation due to the inaccuracy of voting choices and the inaccuracy of vote calculation. An improved DPoS algorithm is introduced in this manuscript to be more effective, versatile, and precise in choosing suitable delegates. Improved DPoS algorithm is made up of three parts. The first is an honor voting system, which yields a collection of delegates phrases for each voting node. The second step is to create an improved voting function that will be used to determine each node's value. The highest number of honor voting, the better the node's chances of becoming a delegate. To complete the voting process, the final step is to determine the divergence level.

### 2.8. The Basic Concept of Improved DPoS Algorithm

According to the literature, the major drawbacks of the public blockchain consensus mechanism are that the distributed ledge techniques are more permissioned and insecure, and its difficulty in creating blocks is considered as a critical limitation to our technological needs. To overcome these issues, the DPoS-based consensus algorithm can solve the security problems because the DPoS algorithm can greatly enhance the authentication and also reduce energy consumption. On the other side, the PoW mechanism greatly reduces the energy consumption because every node has the right to create blocks.

However, achieving fairness by solely employing DPoS is challenging, as it will only allow those with more money to become voters. Furthermore, while employing the PoW alone, the block interval is around 10 minutes, wasting a significant amount of computing power sources. For the power consumption issue, this paper proposes an honor voting mechanism akin to that used in the modern system to reduce the latency of the voting node that causes the consensus to be harmed and replace them. We have considered that there are two types of nodes in the consensus algorithm. The one is the voting node, which creates blocks and takes part in the authentication process. The second node is termed as a honor voting node, and it has special voting privilege. With the special voting privilege, the honor voting node can replace the node when it fails to perform. And, these kinds of proposed nodes are generally considered as the consensus nodes.

### 2.9. The Development of Consensus Mechanism

**Figure 4:**
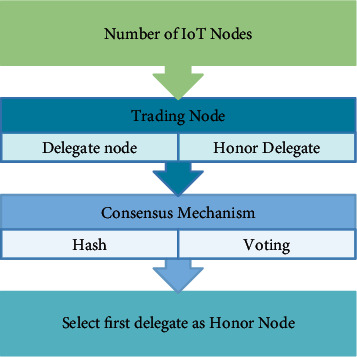
Selecting an honor delegate node in the Improved DPoS Algorithm.

We separate the consensus procedure into two parts. To begin with, utilise the PoW concept to select a set number of suitable nodes from the entire network, and employ stake voting to select consensus nodes. In this network, the top 101 nodes serve as delegate nodes and the remainder nodes serve as honor delegates. The delegate nodes then record the transactions in a block and disseminate it to all consensus nodes for consensus in the second phase. The block will be added to the blockchain if it is successfully verified by more than half of the consensus nodes. The process steps of an honor delegate node selection based on improved DPoS algorithm is shown in Figure 4.

As immediately as the malicious node is discovered, we implement an honor voting method to regenerate it. When a delegate node is discovered to be malicious, it is added to the honor delegates' nodes set, and the rank of all existing delegate nodes is reduced by one. The node in the set of honor delegate nodes with sequence number is moved to the delegate nodes set and ranked last in the delegate nodes set. The malicious node is sorted at the end of the collection of honor delegate nodes, while the identifiers of all the remaining honor nodes are decremented by one.  Algorithm 1 describes the proposed mechanism for honor node selection in the IoT network.

The N number of nodes with the maximum votes can be selected as delegate nodes in improved DPoS, and the shortlist of delegates nodes will be updated every 20 hours, just like in DPoS. The delegate node's reputation and witness identification will be revoked if someone is discovered to have a poor rate of creating blocks or to be engaging in harmful activities.

**Figure 5:**
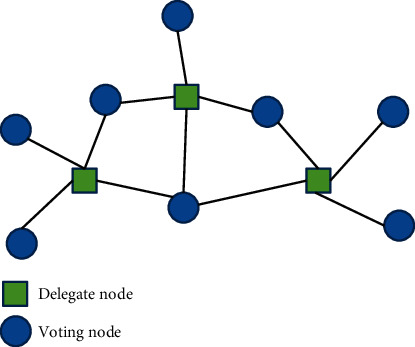
Graph-based delegate nodes selection in consensus mechanism.

The module for choosing consensus networks: all nodes in the public ledger are successfully prepared in order to allocate various tasks to different types of nodes, which are primarily separated into consensus nodes (such as delegate nodes and honor nodes) and transaction network. Trading nodes are responsible for generating transactions, while consensus nodes are responsible for generating and verifying blocks.  Figure 5 shows the delegate node selection using the graph method. The consensus module is responsible for executing the entire process from block creation to block confirmation. The module for degrading malicious nodes is as follows: When a malicious node is discovered, the improved DPoS algorithm switches to a module that replaces honor node and sorts the delegate nodes.

The decentralized network ecosystem is described as a peer-to-peer network made up of all branches in the architecture, with consensus nodes and trade nodes being the two types of nodes in this network. The consensus network is a sub-network ecosystem made up of delegate nodes that will change when the voting number in the improved DPoS algorithm updates. Conversely, the trading sub-network is the network architecture comprising of trading nodes which is not static. The trading nodes update them after each round of selecting the delegate node.

Only during the time between the formation of the current delegate nodes and the beginning of another round of honor node selection will the trading networks be secure. As previously stated, the nodes that participate in the consensus process rather than generating transactions are divided into two categories: delegate node and honor nodes. The trade nodes are accountable for the production, transmitting, and storage of distributed ledger, while the witness nodes take turns recording trades into a block and transmitting it to the other delegate nodes for validation.

## 3. Result Evaluation and Discussion

In this section, we describe the performance assessment of the blockchain-based consensus mechanism for AI-enabled IIoT network. We present the comparison result of the proposed work with existing mechanism such as PoS, PoW, and DPoS in terms of important parameters which includes block creation approach, block generation time, and energy resources. To carry out security performance, we include existing consensus mechanism. Each work represents the artificial intelligence–based big data analysis at the IoT device with decentralized control. The proposed blockchain-based consensus mechanism provides distributed architecture to enhance security and privacy at the IoT device.

The distributed consensus mechanism–based AI-enabled IoT architecture resolved the real-time security issues and also reduced the energy consumption. An improved DPoS algorithm suggested AI-based decentralized IoT network for big data analysis in real-time, and it will overcome the issue of data storage. The proposed mechanism introduces the trading node in which the actual block information is stored. The implementation of big data analytic is developed on the IIoT blocks to evaluate the scalability and robustness of improved DPoS. We presented the performance of the proposed method with existing consensus algorithm research shown in Table 1. According to the security metric, the proposed algorithm provides better results compared with PoS and PoW in terms of energy consumption.

To analyze the data transaction rate of improved DPoS algorithm, we investigate the performance with existing methods such as a PoW, PoS, and DPoS mechanism [43]. The transaction rate of PoW is very low because of its computation time. In PoW, blocks are verified based on the computing power. In the same way, in the case of PoS the blocks are verified through stake methods and it will require more transaction time. In order to reduce the transaction time, DPoS is proposed based on the stake voting mechanism.

Likewise, our proposed algorithm verified through honor delegates which required less time for verification. Therefore, compared with other three mechanisms, our improved DPoS increases the transaction rate.

**Figure 6:**
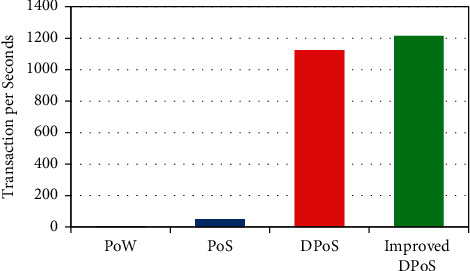
Transaction data rate of various consensus mechanisms.

Figure 6 clearly shows that our improved DPoS algorithm has more TPS than other mechanisms. To reduce the energy resource of the blockchain-based consensus mechanism, we developed an improved DPoS algorithm for IIoT devices. In order to overcome the computing resources problems in the PoW and PoS mechanism of decentralized ledger-based industrialized IoT devices, we propose to combine artificial intelligence and blockchain technology. In the decentralized improved DPoS consensus algorithm implemented to enhance the data privacy and reduce energy resources for big data analysis, the main reason to introduce delegates and honor nodes-based consensus mechanism for IIoT devices is to reduce overall energy consumption, as it will verify the blocks in the idea of stake voting mechanism and also replace malicious nodes through honor delegates. To develop smart contracts between nodes, we used DPoS-based stake voting mechanism, which is denoted as delegates nodes.

**Figure 7:**
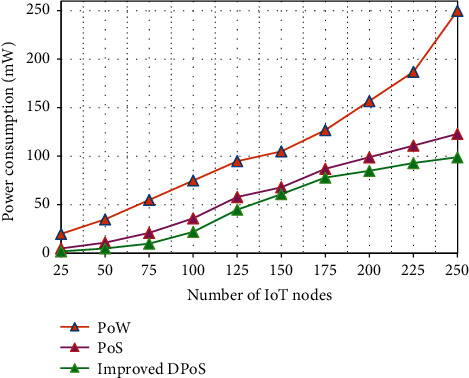
Energy consumption comparison of various consensus mechanisms.

Figure 7 represents the energy consumption analysis of the proposed algorithm and other two existing models. In the PoW mechanism, if the number of blocks increases, the energy consumption also increases because in this algorithm the blocks are created based on the computing power of the particular system. Therefore, the PoW model required more resources than other models. In the case of the proposed algorithm, the blocks are created through stake vote which will be part of the IoT network, as the energy consumption is very less compared to the PoW model.

Finally, the different performance evaluations of the developed blockchain-based AI-enabled IIoT network conforms that an improved DPoS consensus algorithm increases data transaction rate per seconds and reduces the energy resources. Overall, the proposed consensus mechanism is most applicable for AI-based IIoT applications in order to analyze data in a secure manner with less energy resources.

## 4. Conclusion

A combined blockchain and artificial intelligence–based consensus algorithm for big data analysis in IoT applications are introduced in this manuscript. This work aims to develop efficient and reliable IoT data transactions at the industrial level. The suggested DPoS consensus algorithm performance was evaluated using security and energy consumption metrics. An improved DPoS was implemented to blockchain-based AI for decentralized control in IIoT. The experimental analysis is presented to evaluate the performance of the suggested consensus mechanism AI-enabled IIoT applications with distributed and secure big data analytics. In terms of reliability, speed, privacy, and security, the experimental results show the efficiency of the proposed algorithm compared with existing mechanisms. According to the TPS results, the integration of blockchain with artificial intelligence successfully addresses the issues of getting high accuracy, security, and low latency through a decentralized network. The proposed consensus algorithm successfully overcomes the difficulties of accuracy, latency, and security by combining blockchain and artificial intelligence and also addresses the energy consumption issue [44].

## Figures and Tables

**Algorithm 1:**
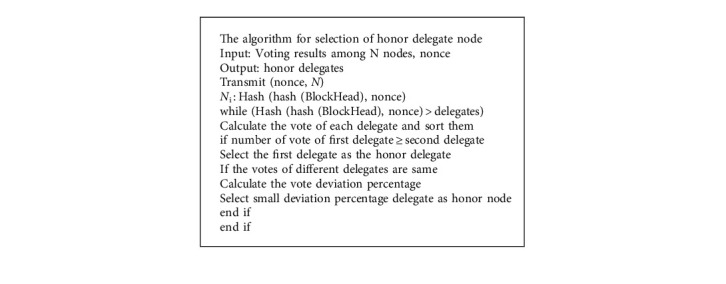
Honor delegates node selection algorithm.

**Table 1:** Performance of various consensus algorithm.

Consensus mechanism	Proof-of-work (PoW)	Proof-of-stack (PoS)	Improved delegate PoS (DPoS)
Mechanism for block generation	Computing power	Stake	Stake votes
Security issues	Constant power	Inactive nodes	Malicious nodes
Energy consumption	Very high	High	Low
Average block generation time	10 min	65 sec	5 sec
Reliability	High	Low	Low
Robustness	High	High	High

## Data Availability

The data used to support the findings of this study are available from the corresponding author upon request.
